# Evidence-Based Programs, Yes—But What About More Program-Based Evidence?

**DOI:** 10.9745/GHSP-D-18-00192

**Published:** 2018-06-27

## Abstract

Policy makers and program managers are better enabled to draw relevant lessons from implementation research and program experience elsewhere when there is richer documentation on what was done and what key contextual factors may have influenced outcomes. Newly developed Program Reporting Standards from WHO provide helpful guidance on what is needed for optimally useful documentation.

See related article by Koek.

## STANDARDS FOR RESEARCH

An important development over the past couple of decades has been the emergence of reporting standards for various types of research. In the early 1990s, journal editors and investigators agreed on standards for reporting randomized controlled trials, issuing the Standardized Reporting of Trials statement.^1^ Over the years since, this has evolved to the Consolidated Standards of Reporting Trials (CONSORT)^2^ and numerous reporting standards have been developed for other types of study and evidence synthesis, many of which can be found on the website of the EQUATOR Network (Enhancing the Quality and Transparency of Health Research) (http://www.equator-network.org/). These standards have not only helped improve reporting of research results but also both raised the bar on quality of the research itself and strengthened guidelines based on evidence synthesis. Nevertheless, this development has been largely driven in response to felt needs of investigators and journal editors rather than those of program managers and policy makers.

## WHY NEW GUIDANCE IS NEEDED FOR PROGRAMMATIC EVIDENCE

Certainly, much is gained from translation of research evidence to practice. But achieving important gains in the complex, messy, real world of programs requires also that we draw evidence from practice.^3^ The World Health Organization (WHO) plays a crucial role providing guidance to country level with regard to both evidence-based interventions addressing key health problems and strategies for delivering them. When guidance is offered on specific biomedical interventions, often we are well served by evidence from randomized controlled trials, and a research-to-practice translation model is helpful. However, for complex interventions and lessons drawn from program experiences, factors other than intervention efficacy also come into play. For policy makers and program managers considering evidence arising from particular program experiences, context and specific details on how an intervention was delivered are crucial in trying to determine to what other circumstances or settings that finding might generalize. Rigorous though their requirements may be in other respects, many research-oriented journals give short shrift to these crucial elements.

Achieving important gains in the complex, messy, real world of programs requires us to draw evidence from practice.

Hales et al. (2016)^4^ describe the process and results of a recent effort to specify reporting standards that more adequately capture the kind of information needed by program managers who are trying to draw actionable lessons from implementation and operations research results. A related effort on reporting standards for complex interventions has carried this further, resulting in Standards for Reporting Implementation Studies (StaRI).^5^ Checklists developed for these standards now include explicit logic pathways, how the tension between fidelity and adaptation is resolved, and adequately rich description of context and its interplay with delivery of the intervention.

## THE WORLD HEALTH ORGANIZATION WEIGHS IN

For policy makers and program managers seeking to draw on what has been learned from programs in other settings that might be relevant to their own, published studies can be ahelpful source of such insights, *providing there is sufficient information on context and how the interventions were delivered*. In addition to studies published in the peer-reviewed literature, good gray literature documentation of such program experiences can be a valuable resource. But to date, although StaRI is a very helpful addition, there hasn't been similar attention to elaborating reporting standards for gray literature. As discussed in the article by Koek et al.^6^ in this issue of GHSP, WHO has recently released a new guidance document, *Program Reporting Standards for Sexual, Reproductive, Maternal, Newborn, Child and Adolescent Health* (PRS), which helps address this gap.^7^ These standards cover both peer-reviewed and gray literature reports documenting implementation and scale up of reproductive, maternal, newborn, child, and related health programs and are intended to result in reporting that can better facilitate cross-program learning on what works (and what doesn't), and under what circumstances. Such learning can aid in decision making on adoption, adaptation, and scale up across diverse settings.

Program managers seeking to draw on learnings from programs in other settings need sufficient information on context and how the intervention was delivered.

Koek et al. acknowledge the value of current research reporting standards but they also point out that for program managers and decision makers existing standards have given insufficient attention to documentation needed on context and implementation details. The new PRS give appropriate attention to issues including:

- Implementation design and logic model
- Context
- The time dimension
- The actors involved in delivery
- Resource requirements
- The planning, piloting, and scale-up process
- Specific activities
- Quality assurance, monitoring, and evaluation
- Coverage and reach achieved
- Adaptations (fidelity vs. flexibility)
- Sustainability
- Strengths and weaknesses
- Lessons learned and implications

More systematic attention to such issues in gray literature reports can make them considerably richer, more useful learning documents for decision makers in other settings who are trying to draw lessons that could be relevant for their own programs.

The new WHO Program Reporting Standards can help make program documentation richer and more useful for decision makers in other settings.

## GHSP SUPPORTS THE PROGRAM REPORTING STANDARDS

At GHSP, we believe these new standards are also useful in specifying the kind of information that can make peer-reviewed implementation and operations research more valuable to program managers. In the Internet age, even hard-copy journals can offer their authors the option of online annexes, to provide richer detail on context and implementation. At the end of the day, we are better able to deliver effective programs and achieve improved population-level health outcomes if we can draw lessons from each other's program experiences.

We fully realize that the PRS represents something of an idealized model. Much useful evidence is developed that does not comprise every element in the standards. And the real world of program implementation can be messy and challenging. Still the elements in the PRS are to a very large extent what we look for in considering what papers to publish. We encourage colleagues in the global health community to use the PRS with vigor.–*Global Health: Science and Practice*
